# Undifferentiated Adipose Tissue Stem Cell Transplantation Promotes Hepatic Regeneration, Ameliorates Histopathologic Damage of the Liver, and Upregulates the Expression of Liver Regeneration- and Liver-Specific Genes in a Rat Model of Partial Hepatectomy

**DOI:** 10.1155/2018/1393607

**Published:** 2018-03-14

**Authors:** Konstantinos G. Apostolou, Ioannis G. Papanikolaou, Charalampos Katselis, Themistoklis Feretis, Dimitrios Kletsas, Manousos M. Konstadoulakis, Maria Lymperi, Angelica A. Saetta, Spiros Tsikalakis, George Agrogiannis, Efstratios Patsouris, George C. Zografos, Apostolos E. Papalois

**Affiliations:** ^1^1st Department of Propaedeutic Surgery, University of Athens, School of Medicine, “Hippocration” Hospital, V. Sofias Avenue 114, 11527 Athens, Greece; ^2^Laboratory of Cell Proliferation & Ageing, National Center for Scientific Research “Demokritos”, Neapoleos 27 Street, 15341 Athens, Greece; ^3^1st Department of Pathology, School of Medicine, University of Athens, Tetrapoleos 18, 11527 Athens, Greece; ^4^Experimental Research Center, ELPEN, Marathonos Avenue 95, 19009 Athens, Greece

## Abstract

**Objective:**

Adipose tissue stem cells (ADSCs) present a promising therapeutic method to alleviate liver failure (LF). The purpose of this prospective study was to evaluate the efficacy of undifferentiated ADSC transplantation on liver regeneration and on the expression of liver regeneration- and liver-specific genes, following 60% partial hepatectomy (PHx).

**Methods:**

Sixty female rats were subjected to PHx and were transplanted with 10^6^ or 2 × 10^6^ ADSCs, either into the portal vein (PV) or into the hepatic parenchyma. Animals of the control group were not transplanted and served as controls. Animals were sacrificed on the 4th, the 7th, or the 15th postoperative day (POD).

**Results:**

The transplanted ADSCs were successfully engrafted into the liver parenchyma and ameliorated the histopathologic damage on the 7th and 15th POD. All transplanted animals demonstrated a significantly higher liver regeneration rate on the 4th and 7th POD, compared with the control group. The expression of hepatocyte growth factor, *α*-fetoprotein, tyrosine aminotransferase, hepatocyte nuclear factor 4a, and cytochrome P450 1A2 was significantly upregulated, compared with the control group.

**Conclusions:**

Although undifferentiated, ADSC transplantation significantly enhanced the liver regeneration process. These findings may be proven clinically valuable, especially in cases of acute LF.

## 1. Introduction

Liver failure (LF) is one of the leading causes of morbidity and mortality worldwide [1]. The only effective treatment so far for acute and chronic LF is liver transplantation [2], with its associated limitations, including the shortage of liver donors and the need for continuous immunosuppression. These facts have prompted the efforts for an alternative treatment of end-stage liver disease (ESLD).

Mesenchymal stem cells (MSCs) present a promising therapeutic method to alleviate ESLD. According to a growing body of evidence in recent years, adipose tissue stem cells (ADSCs), a certain type of MSCs, represent the most promising candidate progenitor cells for transplantation, as they show a stronger commitment to hepatic lineage, as well as higher rates of proliferation, compared to bone marrow mesenchymal stem cells (BM-MSCs) [3, 4].

Despite the encouraging experimental outcomes, many questions still remain, such as type and quantity of transplanted ADSCs, optimal route of administration, and pretreatment or not with growth factors.

In the present prospective study, we aimed to investigate the effect of undifferentiated ADSC transplantation on liver regeneration, as well as on the expression of liver-specific genes, in a rat model of partial hepatectomy (PHx), in relation to the number and their route of administration.

## 2. Materials and Methods

### 2.1. Animals

One hundred Wistar rats of conventional microbiological status (*n* = 90 female, *n* = 10 male), weighing 190–260 g, were purchased from the same breeder (Democritus, Agia Paraskevi, Greece). All rats were grouped and housed in type IV cages with 400 cm^2^ floor area per rat, with a controlled environment of 12 h : 12 h light-dark cycle. All animals had ad libitum access to food and water. They were allowed to acclimate to the laboratory conditions for at least one week prior to the experiment. All studies carried out at the Experimental Research Center, ELPEN conform to the Presidential Decree 56/2013 for the Protection of Animals used for Scientific Purposes. Male Wistar rats were used as ADSC donors, while the female rats as the recipients of ADSCs.

### 2.2. Experimental Design

Female rats were randomly allocated to one of six different experimental groups. Control group (*n* = 15) underwent 60% PHx, without transplantation. Sham-operated group (*n* = 15) underwent a midline laparotomy with incision of the liver ligaments, followed by abdominal closure. Groups A and B (*n* = 15/group) underwent 60% PHx with subsequent administration of 10^6^ and 2 × 10^6^ ADSCs into the portal vein (PV), respectively. Groups C and D (*n* = 15/group) underwent 60% PHx with subsequent administration of 10^6^ and 2 × 10^6^ADSCs into the hepatic parenchyma, respectively. Group N (*n* = 90), although not belonging to experimental groups, represents the preoperative values of all animals. Each experimental group was subdivided into three subgroups (*n* = 5/subgroup), depending on the postoperative day (POD) of sacrifice (Table 1).

### 2.3. Isolation and Culture of Rat ADSCs

White adipose tissue was collected from rats and was immediately transferred to the laboratory at 4°C. The tissue was washed with phosphate-buffered saline (PBS), minced using two scalpels, and then was digested in crude collagenase (1 mg/ml DMEM) for 30 min at 37°C. Subsequently, the digest was centrifuged (200*g* for 5 min) to discard the supernatant and the pellet was resuspended in DMEM/10% FBS/1% penicillin/streptomycin and transferred to a culture flask. After an overnight incubation, the medium was changed as to remove the nonadherent cells and the attached cells were further cultured in the same medium.

### 2.4. Surgical Procedure and Euthanasia

**Figure 1:**
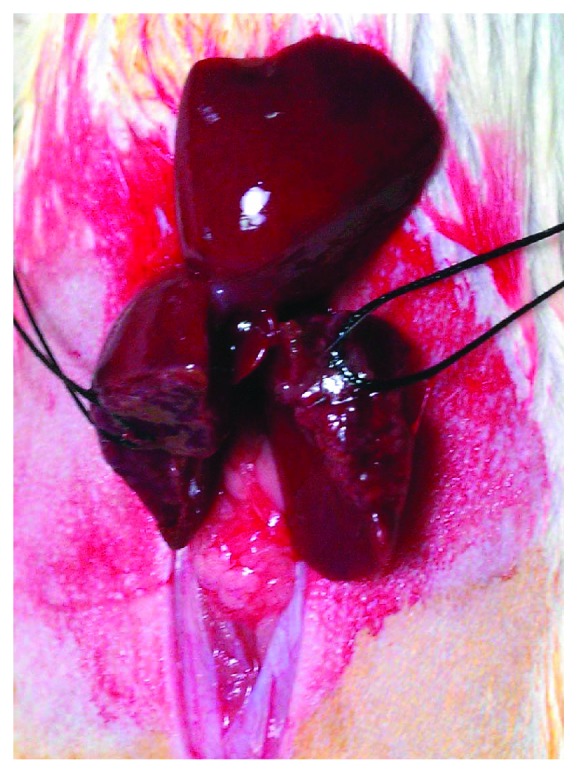
60% partial hepatectomy (PHx) was performed, by dividing the left lateral lobe (LLL), and median lobe (ML) of the liver near the origin of their vasculature by using electrocautery, followed by suture ligation and resection.

**Figure 2:**
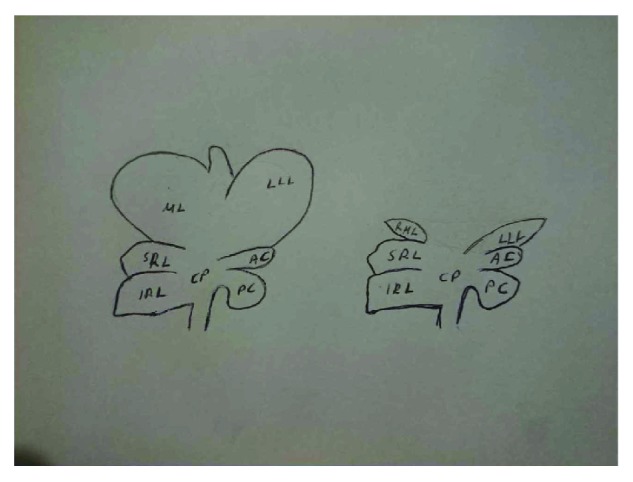
Indicating the normal liver anatomy of the rat (on the left) and the liver anatomy after our surgical technique of nearly totally resecting the left lateral lobe (LLL) and median lobe (ML) of the liver (on the right), thus achieving a 60% partial hepatectomy.

**Figure 3:**
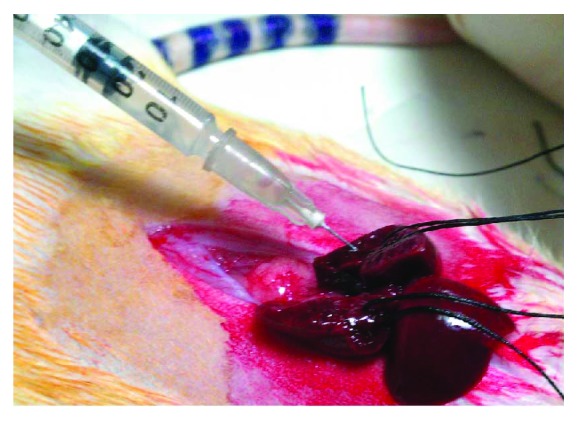
10^6^ ADSC transplantation into the remnants of the resected liver lobes (LLL and ML), with the use of a 30-gauge needle, at a dialysis of 10^6^ ADSCs in 0.2 ml of saline.

All rats had no access to food and water for the last 4 hours before the surgical procedure. General gas anesthesia was induced and maintained by a mixture of O_2_ and N_2_O and isoflurane (Forenium®, 4% for induction and 2% for maintenance). All interventions were performed under sterile conditions. A midline laparotomy was performed, followed by incision of liver ligaments. The intestinal loops were shifted towards the left side, while the left lateral lobe (LLL) and the median lobe (ML) of the liver were shifted cranially. Rats were subjected to 60% PHx, by dividing the LLL and ML near the origin of their vasculature using electrocautery, followed by suture ligation and resection (Figures 1 and 2). The resected liver specimen was immediately weighed to estimate the resected liver mass, as well as the percentage of PHx performed, as the liver mass represents approximately 5% of the rat's total body weight [5]. Following PHx, gentle dissection was carried out, and the PV was exposed posteriorly and laterally to the hepatic artery (HA) and common bile duct (CBD). ADSCs were administered either into the PV or into the remnants of the resected liver lobes, with the use of a 30-gauge needle, at a dialysis of 10^6^ ADSCs in 0.2 ml of saline (Figure 3). Finally, 1 ml NaCl 0.9% was administered intraperitoneally and the abdomen was closed in a continuous one-layer fashion. Next, rats were placed under heat-producing lamps to recover from anesthesia.

**Figure 4:**
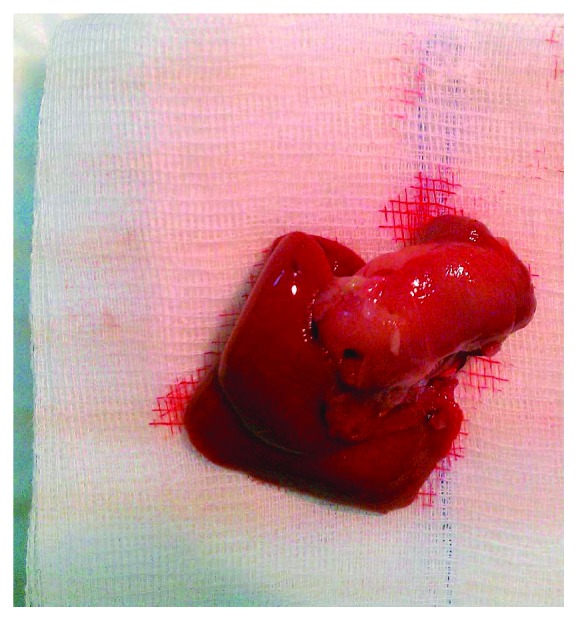
The harvested regenerated liver at the time of euthanasia.

Rats of each group were randomly allocated to be sacrificed either on the 4th or on the 7th day or on the 15th POD. Rats were anesthetized before euthanasia, followed by animal weighing. All rats had no access to food and water for the last 4 hours before euthanasia. A midline laparotomy was performed and blood samples were taken from the inferior vena cava (IVC), followed by harvesting of the liver, which was also weighed (Figure 4). Four representative tissue samples from the nearly totally resected liver lobes as well as four tissue samples from the nonresected lobes were harvested. Half of the tissue was fixed in 4% buffered formaldehyde, embedded in paraffin, and routinely stained with haematoxylin and eosin (H&E staining), while the other half was immediately transferred in liquid nitrogen and then stored at −80°C for future RNA extraction.

### 2.5. Peripheral Blood Sample Analysis

The levels of platelets (PLT), aspartate aminotransferase (AST), alanine aminotransferase (ALT), gamma-glutamyltransferase (GGT), alkaline phosphatase (ALP), albumin (ALB), prothrombin time (PT), (INR), total proteins (PR), total bilirubin (TBIL), direct bilirubin (DBIL), indirect bilirubin (IBIL), and phosphorus were measured in peripheral blood sample, in a single center, by standard laboratory methods.

### 2.6. Liver Regeneration Rate

Liver regeneration rate (%) was calculated on the day of sacrifice, using the following equation: 100 × {*C*–(*A* − *B*)}/*A*, where *A* is the estimated total liver weight at the time of PHx, which represents approximately 5% of the rat's total body weight [5], *B* is the weight of the excised liver, and *C* is the weight of the harvested regenerating liver at the time of sacrifice [6, 7].

### 2.7. Body Weight Assessment

Total body weight was measured prior to the surgical procedure (initial weight (IW)) as well as at the time of sacrifice, with the rats under general gas anesthesia and still alive (preeuthanasia weight (PEW)). Preeuthanasia weight (PEW%) was calculated, using the following equation: PEW = 100 × (PEW − IW)/IW.

### 2.8. Fluorescence In Situ Hybridization

10 *μ*l of ZytoLight Rat Y/12 Fluorescence in Situ Hybridization (FISH) Y- chromosome probe (ZytoVision GmbH) was applied onto each individual deparaffinized liver section of 4 *μ*m thickness, according to the guidelines of the manufacturer. Sample material was evaluated by fluorescence microscopy, with filter sets for the wavelength ranges applied.

### 2.9. Histological Analysis

Multiple 4 *μ*m sections were scored blindly, by two independent observers, for the following parameters: sinusoidal congestion, vacuolization of hepatocyte cytoplasm, parenchymal necrosis, and inflammation, with the total score representing the sum of all parameters for each individual animal. Each parameter was graded numerically as follows: congestion, vacuolization, and inflammation: 0 = none, 1 = minimal, 2 = mild, 3 = moderate, and 4 = severe. The numerical graduation for necrosis was as follows: 0 = nonnecrotic cells, 1 = single-cell necrosis, 2 ≤ 30% necrosis, 3 ≤ 60% necrosis, and 4 ≥ 60% necrosis.

### 2.10. RNA Extraction and Reverse Transcription Quantitative Real-Time PCR (RT-qPCR)

Total RNA was extracted using NucleoSpin® RNA Plus (Macherey-Nagel GmbH & Co. KG, Germany). RNA concentration and quality were determined using the NanoDrop 2000 UV-Vis Spectrophotometer (Thermo Fisher Scientific, DE, USA). 250 ng of total RNA was converted to cDNA using Superscript II RT-PCR kit (Invitrogen Life Technologies, CA, USA). Liver regeneration- as well as liver-specific genes (hepatocyte growth factor (HGF), *α*-fetoprotein (AFP), albumin (ALB), glypican 3 (GPC3), tyrosine aminotransferase (TAT), hepatocyte nuclear factor 4A (HNF-4a), and cytochrome P450 1A2 (CYP1A2)) relative mRNA expression levels were determined by reverse transcription quantitative real-time PCR (RT-qPCR), by using the 2^−ΔΔCT^ method, on LightCycler® 480 System (Roche Diagnostics GmbH, Germany), using Maxima® SYBR Green/ROX kit (Thermo Fisher Scientific, DE, USA). The samples were run at least in duplicates, and for each sample, the mean Cp value was calculated. As an appropriate endogenous control, the GAPDH gene was selected according to the literature [8, 9]. Three pool samples (control group) were prepared in total, respective to the day of sacrifice, containing all individuals of each subset, and each sample was analyzed with the time-matching pool sample as a calibrator. Relative expression was then assessed by LightCycler 480 Software, Version 1.5 (Roche Diagnostics GmbH, Germany). Sequences of gene- and rat-specific primers used are depicted in Table 2.

### 2.11. Statistical Analysis

Statistical analysis was done with IBM SPSS Statistics ver. 20 (SPSS Inc., Chicago, IL, USA), by a statistician specializing in medical statistics. The level of statistical significance was set at 5% (*α* = 0.05).

Analysis of variance (with pairwise post hoc Bonferroni tests) was used to compare the mean values of all parameters by time period (4 days, 7 days, and 15 days) for each group (CN, S, A, Β, C, and D), respectively. Each time the assumption of homogeneity of variances, which is crucial for ANOVA, was checked with Levene's test.

## 3. Results

### 3.1. Tracing of Transplanted ADSCs

**Figure 5:**
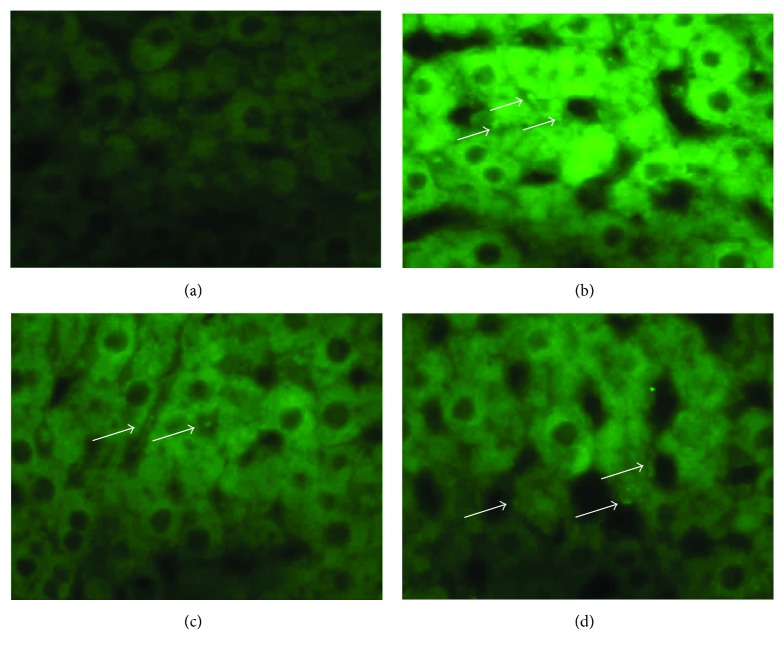
(a–d) Fluorescent in situ hybridization (FISH) of the Y chromosome, with fluorescein stain used for nuclear staining. (a) Animal of the control group, in which no signals are present. (b) Localization of the intraportally transplanted ADSCs in the host liver on the 4th POD (as depicted by arrows). (c and d) Localization of the intraparenchymaly transplanted ADSCs in the host liver on the 7th and 15th POD, respectively (as depicted by arrows).

The expression of the rat Y chromosome was observed in the liver parenchyma of all transplanted rats of all POD of sacrifice, irrespective of the number or the route of transplantation, whereas it was not observed in nontransplanted animals, thus demonstrating the successful transplantation and localization of ADSCs (Figure 5).

### 3.2. Analysis of Histopathologic Damage

**Figure 6:**
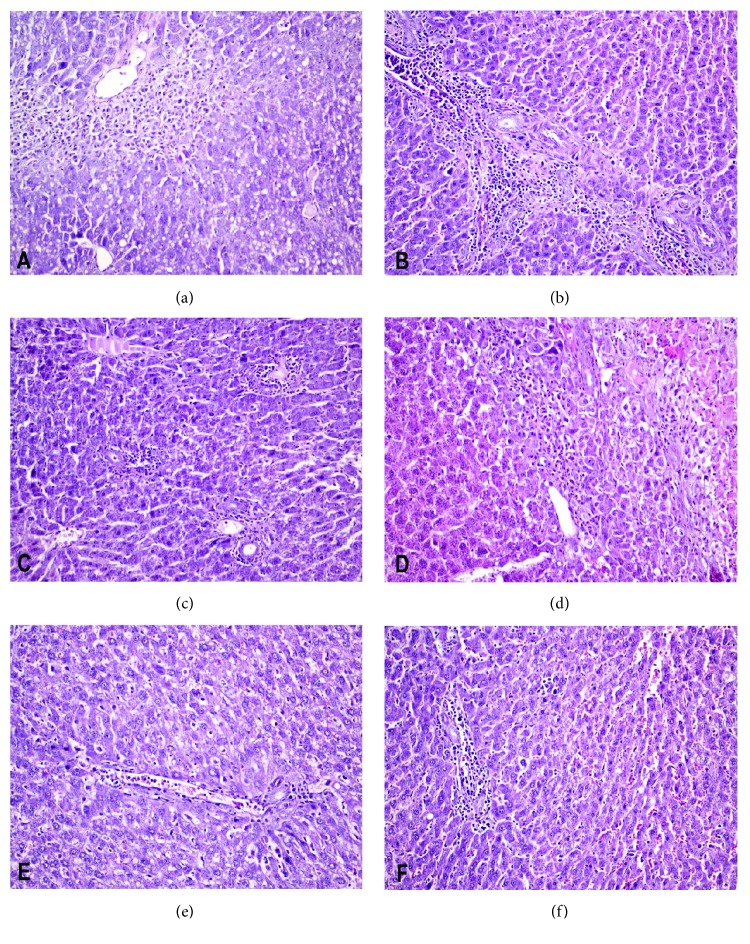
Histological analysis of the liver. (a, c, and e) Indicating the presence of severe congestion, vacuolization, inflammation, and necrosis in an animal of the CN_2_ subgroup at the 7th postoperative day (a), which is statistically significantly improved in a transplanted animal of the B2 subgroup (c) and resembles normal liver histology in a transplanted animal of the C2 subgroup (e). (b, d, and f) Indicating the presence of moderate congestion, vacuolization, and inflammation in an animal of the CN3 subgroup at the 15th postoperative day (b), which is statistically significantly improved in a transplanted animal of the B3 subgroup (d) and resembles normal liver histology in a transplanted animal of the D3 subgroup (f).

Regarding the animals that were sacrificed on the 4th POD, no statistically significant differences in the total score as well as in each individual parameter were identified between the transplanted as well as between the transplanted and the nontransplanted animals. On the 7th and 15th POD, however, a significantly improved total score was observed in the subgroups B_2_ (*p* = 0.044) and C_2_ (*p* = 0.044), as well as in the subgroups A_3_ (*p* = 0.047) and B_3_ (*p* = 0.047), compared with the respective control animals, with no significant differences between the transplanted groups (Table 3) (Figure 6).

### 3.3. ADSC Transplantation Promotes Hepatic Regeneration

**Figure 7:**
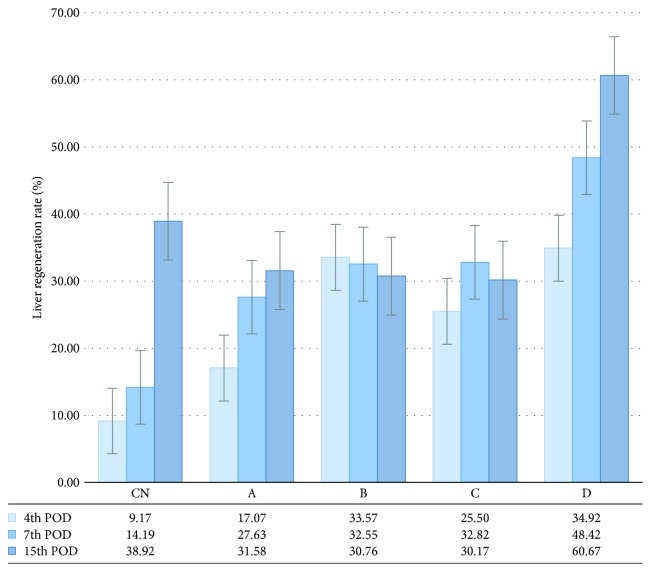
Liver regeneration rate (%), as calculated on the POD^1^ of sacrifice, using the equation: 100 × {*C* − (*A* − *B*)}/*A*, where *A* is the estimated total liver weight at the time of PHx^2^, *B* is the weight of the excised liver, and *C* is the weight of the harvested regenerating liver at the time of sacrifice. The vertical lines indicate the standard error of the mean (SEM). Liver regeneration rate was not calculated for the sham group, as these animals did not undergo PHx. The experimental groups are depicted in Table 1 (experimental design). ^1^POD: postoperative day; ^2^PHx: partial hepatectomy.

The mean percentage of PHx that all rats were subjected to was 59.7%, without any significant difference between the transplanted and the nontransplanted groups. A significantly greater liver regeneration rate was observed in the subgroups B_1_ (*p* = 0.022) and D_1_ (*p* = 0.014), as well as in the subgroup D_2_ (*p* = 0.021), compared with the respective control subgroups, without any significant differences between the transplanted animals of the same POD. On the 15th POD, no significant differences in the regeneration rate were observed between the transplanted animals, as well as between the transplanted and the respective control animals (Figure 7).

### 3.4. Body Weight as a Nutritional Status Parameter

**Figure 8:**
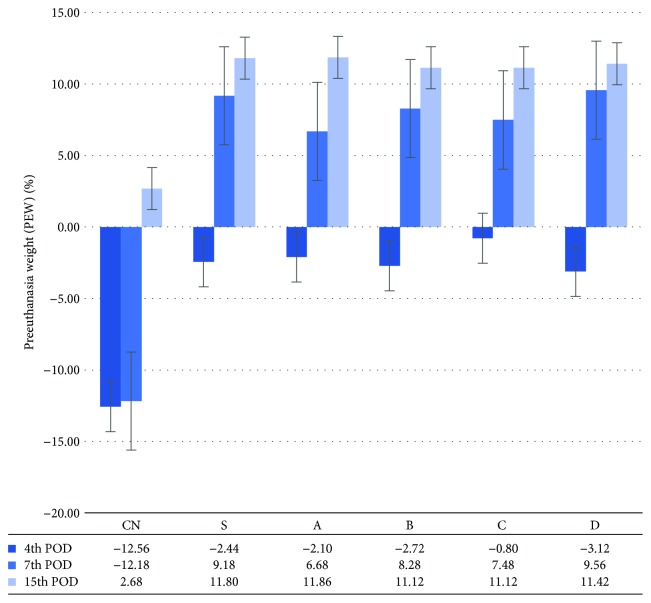
Preeuthanasia weight (%) (PEW (%)), as calculated on the POD^1^ of sacrifice, using the equation: preeuthanasia weight (PEW) = 100 × (PEW − IW)/IW, where PEW is the weight at the time of sacrifice and IW is the initial weight prior to PHx^2^. The vertical lines indicate the standard error of the mean (SEM). The experimental groups are depicted in Table 1 (experimental design). ^1^POD: postoperative day. ^2^PHx: partial hepatectomy.

A significantly greater PEW was observed in all the transplanted subgroups of the 4th and 7th POD, compared with the respective control animals, whereas no significant differences were observed between the transplanted animals. On the 15th POD, no significant differences in PEW were observed between the transplanted, as well as between the transplanted and the nontransplanted animals (Figure 8).

### 3.5. Peripheral Blood Sample Markers of Liver Function

The levels of AST and ALT in the transplanted animals decreased, compared with their control counterparts, without though any significant difference on the 4th and 7th POD. On the 15th POD, a nonsignificant increase in the levels of AST and ALT was recorded, compared with the levels on the 4th and 7th POD (Tables 4 and 5).

Significantly improved PT and INR values were demonstrated in the transplanted animals of all POD of sacrifice, compared with their control counterparts, with no significant differences between the transplanted ones (Table 5). The PLT number increased in all animals of the 4th and the 7th POD, compared with the preoperative values, with significant differences specifically identified, without, however, any significant difference between the transplanted and the nontransplanted animals (Table 4). Although the serum levels of ALB were slightly higher in the control group, compared with the majority of the transplanted groups of all POD, significant differences were specifically identified on each POD (Table 5).

The levels of GGT, ALP, TBIL, DBIL, IBIL, and PR were evaluated in blood serum, as markers of function of hepatocytes and cholangiocytes, and the significant differences are depicted in Tables 6 and 7. Although the serum levels of phosphorus in all the transplanted animals of all POD were greater than the preoperative ones, they were still decreased on the 4th and 7th POD, compared with their control counterparts, without though any significant difference.

### 3.6. Expression Levels of Liver Regeneration- and Liver-Specific Genes

**Figure 9:**
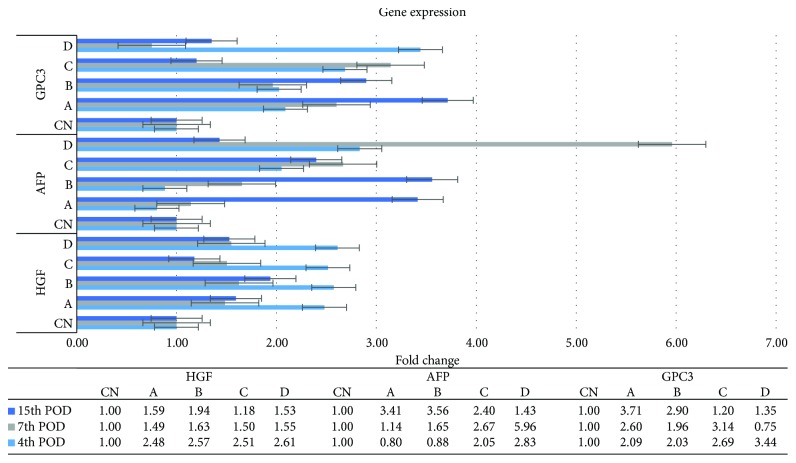
HGF, AFP, and GPC3 mRNA expression levels, as determined by reverse transcription quantitative real-time PCR (RT-qPCR), by using the 2^−ΔΔCT^ method. The horizontal lines indicate the standard error of the mean (SEM). The experimental groups are depicted in Table 1 (experimental design).

All the transplanted animals showed a significantly higher expression ratio of the HGF gene on the 4th and 7th POD of sacrifice, compared with the respective control animals. On the 15th POD, the expression of the HGF gene was nearly identical to that of the 7th POD, without, though, any significant difference (Figure 9).

The expression ratio of the AFP gene was significantly higher in the D_1_ subgroup, compared with the CN_1_ (*p* = 0.04), A_1_ (*p* = 0.02), and B_1_ (*p* = 0.03) subgroups, as well as in the D_2_ subgroup, compared with the CN_2_ subgroup (*p* = 0.04). A completely different pattern was observed on the 15th POD, with the intraportally transplanted animals demonstrating a significantly higher ratio, compared with the control animals, without other significant differences (Figure 9).

**Figure 10:**
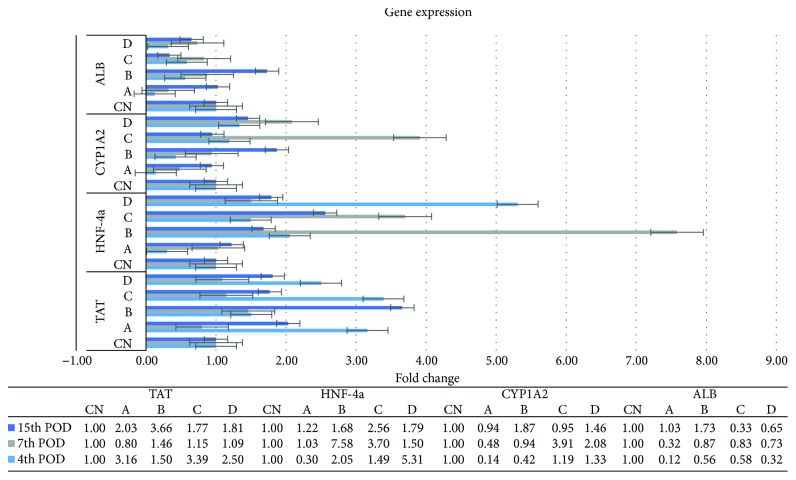
TAT, HNF-4a, CYP1A2, and ALB mRNA expression levels, as determined by reverse transcription quantitative real-time PCR (RT-qPCR), by using the 2^−ΔΔCT^ method. The horizontal lines indicate the standard error of the mean (SEM). The experimental groups are depicted in Table 1 (experimental design).

The expression profile of TAT showed two peaks, on the 4th and on the 15th POD. Except for the B_1_ subgroup, all the transplanted animals of the 4th and 15th POD demonstrated an increased expression of the TAT gene, compared with the respective control subgroups, with significant differences recorded only on the 15th POD (Figure 10).

On the 4th POD, the expression of the HNF-4a gene was higher in nearly all the transplanted animals, compared with their control counterparts, with significant differences identified between the D_1_ and the CN_1_ (*p* = 0.04) and A_1_ (*p* = 0.02) subgroups. A different pattern was recorded on the 7th and the 15th POD, with the B_2_, C_2_, and C_3_ subgroups showing a higher but not significant ratio, compared with their control counterparts (Figure 10).

Despite the differences in the expression ratio of the CYP1A2 gene in all animals of all POD of sacrifice, no statistically significant differences were demonstrated (Figure 10).

Although the GPC3 gene was highly expressed in nearly all the transplanted animals of all POD, compared with their control counterparts, a significant difference was solely recorded between the A_3_ and the CN_3_ subgroup (*p* = 0.04) (Figure 9).

A significantly lower expression ratio of the ALB gene was demonstrated in all the transplanted animals of the 4th and 7th POD, compared with their control counterparts. On the 15th POD, however, the B_3_ subgroup showed an increased, but not significant, expression, compared with the control counterparts, with significant differences identified between the B_3_, and C_3_ (*p* = 0.001) and D_3_ (*p* = 0.009) subgroups (Figure 10).

## 4. Discussion

ADSCs are in abundance and may be harvested with the use of minimally invasive procedures [10]. They secrete growth factors and cytokines associated with liver regeneration, such as HGF, vascular endothelial growth factor (VEGF), and interleukin-6 (IL-6) [10–13].

It was initially thought that MSCs' therapeutic potential originated from their pretransplantation hepatic differentiation. On the other hand, undifferentiated MSCs are less receptive to oxidative stress and thus more likely to survive the initial hypoxic phase following transplantation [14]. Moreover, stem cells could act either in a paracrine or in an endocrine fashion, thus affecting adjacent cells by secreting growth factors and cytokines [11, 13]. This attenuation of the promoted liver regeneration process in the transplanted animals of the present study after the 7th POD could be explained by the fact that the liver regeneration process is a well-orchestrated phenomenon, which in rats is completed within 7 days following PHx; thus, no difference in the liver regeneration rate should be expected from the 7th POD and onwards. Our findings are also in accordance with several other reports, which have demonstrated that circulating stem cells mobilized to the injured liver and then they began to proliferate and restored liver histology and function [7, 15–24].

Despite the lack of statistical significance, possibly due to the small number of animals in each experimental subgroup, the decreased levels of AST and ALT in the transplanted animals of the 4th and 7th POD, compared with the respective control subgroups, are further supported in the literature [25, 26]. On the other hand, several factors may be responsible for the increase in the levels of AST and ALT on the 15th POD, including the neovascularization phenomenon, which may lead to an increased outflow of accumulated proteins of hepatocellular damage, such as AST and ALT [26]. This phenomenon may be further triggered by the enhancement of the liver regeneration process in the transplanted animals and necessitates further investigation.

HGF is present in liver matrix and is mainly produced by the stellate cells but also by endothelial cells of the liver [27, 28]. Given its properties as a direct mitogen for hepatocytes as well as its activation early in the liver regeneration process, it is considered as the initiator of the liver regeneration and the most irreplaceable factor of this process [29]. Considering that HGF levels increase as a response to PHx, as well as that active HGF is consumed from the intrahepatic stores in the first 3 hours after PHx, followed by de novo HGF synthesis, our results demonstrate that the transplanted ADSCs promoted the expression of the HGF gene, which in turn lead to an upregulated liver regeneration rate, as also described in other studies [7].

AFP is expressed in hepatic oval cells, as well as in cells differentiating towards the hepatic lineage [30]. In maturing hepatocytes, the expression of AFP gradually declines; thus, AFP is consequently used as a marker of early hepatic differentiation [31]. The significantly increased expression of the AFP gene in the majority of the transplanted animals of all POD of sacrifice in the present study is indicative of an upregulated differentiation process of the regenerating cells, including the ADSCs, towards the hepatic lineage. One step further, differentiated cells still expressed genes of their former early differentiation state, even on the 15th POD, as also described by other studies [8, 32].

Previous reports have demonstrated that an increase in TAT expression levels precedes its increase in activity, manifested as a peak at 8 to 18 hours following PHx [33, 34]. There is evidence that the increase in the enzyme's activity is due to de novo enzyme synthesis [33]. Our results indicate a positive effect of ADSC transplantation on the expression of the TAT gene, throughout the entire follow-up period, which due to the lack of further studies with a follow-up period of more than 7 days following PHx requires further investigation.

HNF-4a was initially thought of as stably expressed in hepatocytes, without any significant changes during liver regeneration; therefore, it was used as a marker of mature hepatocytes [35]. Contrary to that, the results of the present study are in agreement with the data published by other studies [36], demonstrating that HNF-4a expression is determined by the liver regeneration and differentiation process and is significantly upregulated during the intermediate phase of liver regeneration [34].

Cytochrome 1A2 is responsible for the metabolism of drugs and toxic compounds. It has been demonstrated that hepatic progenitor cells do not express many of the P450 isoforms, whereas the increase of their expression coincides with the differentiation process [37, 38]. Our findings are indicative of an upregulated differentiation process of the regenerating cells, including the ADSCs, towards the hepatic lineage in the intraparenchymal transplanted animals, which attenuates after the 7th POD, possibly due to the already complete or near-complete liver regeneration process, as well as restoration of the synthetic and metabolic activity of the regenerated liver.

Several reports have demonstrated that MSCs, either pretreated or not with growth factors, transplanted in animals with liver injury, expressed the ALB gene, which is a gene expressed in mature hepatocytes [39]. In the present study, ALB followed a time-dependent expression pattern, as also demonstrated in other studies [39], in which the lower expression levels in the transplanted animals on the 4th and 7th POD were followed by a slight overexpression of the ALB gene on the 15th POD in the intraportally transplanted animals, compared with the respective control animals. Considering, however, the expression pattern of the ALB gene and the serum levels of ALB, it seems that the higher expression ratio of the gene in the intraportally transplanted animals of the 15th POD is not translated into higher serum levels of ALB. Several changes in the posttranscription level may be responsible for this phenomenon and need further investigation.

Multiple studies have focused on the role of GPC3 in liver regeneration. It has been implicated that GPC3 may be a negative regulator of liver regeneration and hepatocyte proliferation [40]. However, our results indicate that GPC3 expression is upregulated throughout the entire postoperative follow-up period, possibly controlling the liver regeneration process, through its negative regulatory action. However, more light has to be shed on its exact mechanism of action during liver regeneration, which still remains elusive.

The optimal route of transplantation as well as the optimal number of transplanted MSCs is still a matter of debate. Some studies support the systemic transplantation of MSCs, via a peripheral vein, as a better route of transplantation, compared with the PV or the intrahepatic administration [17], while other studies support the PV as the optimal route [15, 41]. In the present study, although the IH administration of ADSCs rendered the best results in promoting the liver regeneration process, compared with the respective control group on the 4th and 7th POD, no other specific significant differences were identified among the transplanted animals, as for the number and route of administration of ADSCs, a finding also supported by several other studies [42, 43].

In the context of the limited time in treating and saving a patient suffering from ALF, the results of the present study are of particular value, when applied to a clinical setting. The cornerstone of our study is the successful transplantation of ADSCs, without a previous in vitro differentiation towards the hepatic lineage, which needs a considerable amount of time to complete, ranging from 2 weeks to several months [15, 18, 21, 44–46]. This results in a fast and definitive enhancement of the liver regeneration process, as well as in an upregulation of the synthetic ability of the liver, without sacrificing any time in the differentiation process, which may be proven clinically valuable.

Although the PHx model in rats is a well-studied area [29], the present study demonstrated a new surgical approach for PHx, by dividing the LLL and ML near the origin of their vasculature using electrocautery, followed by suture ligation and resection, instead of totally resecting these two lobes. This approach allowed us to administer the ADSCs into the liver parenchyma of the nearly totally resected lobes, instead of administering them into the parenchyma of the intact remaining lobes. Moreover, this approach allowed us, at the time of euthanasia, to harvest representative tissue samples from both the nearly totally resected liver lobes, where the histological and regeneration changes are more likely to be more evident, and from the intact lobes. On the other hand, the major limitation of the present study is the fact that it compared the outcomes between experimental groups that were transplanted via specific routes and with specific number of ADSCs in each case, instead of comparing the outcomes between all the available transplantation routes as well as numbers of transplanted ADSCs, which would shed more light on the optimal route and number of transplanted ADSCs. However, this fact might have challenged a complex study, with a huge number of animals and statistical comparisons, as well as abundant human and financial resources required.

## 5. Conclusion

An in vitro differentiation of the ADSCs towards the hepatic lineage is not a prerequisite for a successful outcome, as the transplanted undifferentiated ADSCs managed to successfully engraft into the liver parenchyma and promote the liver regeneration process. Moreover, they ameliorated the histopathologic damage of the liver and at the same time upregulated the expression of liver regeneration- and liver-specific genes, irrespective of the number and route of transplantation. This promotion of the liver regeneration process, without sacrificing any time in a pretransplantation differentiation process, may be proven valuable in the clinical context, especially in cases of acute LF, and opens new horizons in the treatment of ESLD.

## Figures and Tables

**Table 1:** Experimental design.

Experimental group	Subgroup	Number of animals/subgroup	Subgroup identification number	POD^1^ of sacrifice	Route of ADSC^2^ transplantation	Number of transplanted ADSCs
Control group	4 days	5	CN_1_	4th	—	—
	7 days	5	CN_2_	7th	—	—
	15 days	5	CN_3_	15th	—	—

Sham group	4 days	5	S_1_	4th	—	—
	7 days	5	S_2_	7th	—	—
	15 days	5	S_3_	15th	—	—

Group A	4 days	5	A_1_	4th	PV^3^	10^6^
	7 days	5	A_2_	7th	PV	10^6^
	15 days	5	A_3_	15th	PV	10^6^

Group B	4 days	5	B_1_	4th	PV	2 × 10^6^
	7 days	5	B_2_	7th	PV	2 × 10^6^
	15 days	5	B_3_	15th	PV	2 × 10^6^

Group C	4 days	5	C_1_	4th	HP^4^	10^6^
	7 days	5	C_2_	7th	HP	10^6^
	15 days	5	C_3_	15th	HP	10^6^

Group D	4 days	5	D_1_	4th	HP	2 × 10^6^
	7 days	5	D_2_	7th	HP	2 × 10^6^
	15 days	5	D_3_	15th	HP	2 × 10^6^

Indicating the six different experimental groups as well as the three subgroups in each experimental group, according to the number and route of transplantation of ADSCs and the postoperative day of euthanasia. ^1^POD: postoperative day; ^2^ADSCs: adipose tissue stem cells; ^3^PV: portal vein; ^4^HP: hepatic parenchyma.

**Table 2:** Sequences of primers used for reverse transcription quantitative real-time PCR (RT-qPCR).

Gene	Forward	Reverse
AFP^1^	AGAAAACAGGGCGATGTCCA	TGCCTTGTCATACTGAGCGG
GPC3^2^	TAAAAGTCGCCCGTGTCGAA	ATGTAGCCTGGCAAAGCACT
HGF^3^	CCCTATTTCCCGTTGTGAAGGA	ACCATCCACCCTACTGTTGTTT
TAT^4^	GATTTTGGCAGTGGCTGAAAGG	GAACATTGGTGCTGAGGTTGG
ALB^5^	AAGAGAAAGCACTGGTCGCA	GGGGAATCGCTGGCTCA-TAC
HNF-4a^6^	AGGATGAAGAAGTTGCCCCC	GATGTGTCTGGTGGGTCCTG
CYP1A2^7^	CATCCTTTGTCCCCTTCACCA	GGTCTTTCCACTGCTTCTCATC
GAPDH^8^	CTCTCTGCTCCTCCCTGTTC	TACGGCCAAATCCGTTCACA

The sequences of primers (forward and reverse) used for reverse transcription quantitative real-time PCR (RT-qPCR). ^1^AFP: *α-*fetoprotein; ^2^GPC3: glypican 3; ^3^HGF: hepatocyte growth factor; ^4^TAT: tyrosine aminotransferase; ^5^ALB: albumin; ^6^HNF-4a: hepatocyte nuclear factor 4a; ^7^CYP1A2: cytochrome P450 1A2; ^8^GAPDH: glyceraldehyde 3-phosphate dehydrogenase (GAPDH was used as an endogenous control).

**Table 3:** Liver histological analysis scoring.

Animal	Subgroup ID^1^	Congestion	Vacuolization	Necrosis	Inflammation	Total score^2^
CN1-1	CN1	1	0	0	0	**1**
CN1-2	CN1	1	0	1	1	**3**
CN1-3	CN1	1	1	1	1	**4**
CN1-4	CN1	1	0	0	0	**1**
CN1-5	CN1	1	1	1	3	**6**
CN2-6	CN2	1	1	0	0	**2**
CN2-7	CN2	1	1	1	0	**3**
CN2-8	CN2	1	1	0	1	**3**
CN2-9	CN2	0	0	0	0	**0**
CN2-10	CN2	2	2	3	3	**10**
CN3-11	CN3	1	1	1	1	**4**
CN3-12	CN3	1	0	1	0	**2**
CN3-13	CN3	0	1	0	0	**1**
CN3-14	CN3	0	0	1	0	**1**
CN3-15	CN3	1	0	1	0	**2**
S1-1	S1	0	0	0	0	**0**
S1-2	S1	0	0	0	0	**0**
S1-3	S1	0	0	0	0	**0**
S1-4	S1	0	0	0	0	**0**
S1-5	S1	0	0	0	0	**0**
S2-6	S2	0	0	0	0	**0**
S2-7	S2	0	0	0	0	**0**
S2-8	S2	0	0	0	0	**0**
S2-9	S2	0	0	0	0	**0**
S2-10	S2	0	0	0	0	**0**
S3-11	S3	0	0	0	0	**0**
S3-12	S3	0	0	0	0	**0**
S3-13	S3	0	0	0	0	**0**
S3-14	S3	0	0	0	0	**0**
S3-15	S3	0	0	0	0	**0**
Α1-1	A1	1	0	2	2	**5**
Α1-2	A1	0	3	1	1	**5**
Α1-3	A1	0	3	1	1	**5**
Α1-4	A1	0	1	0	0	**1**
Α1-5	A1	1	3	1	1	**6**
Α2-6	A2	0	1	0	0	**1**
Α2-7	A2	0	0	0	0	**0**
Α2-8	A2	0	0	0	0	**0**
Α2-9	A2	1	0	1	2	**4**
Α2-10	A2	0	0	0	0	**0**
Α3-11	A3	0	0	0	0	**0**
Α3-12	A3	0	0	0	0	**0**
Α3-13	A3	0	0	0	0	**0**
Α3-14	A3	0	0	0	0	**0**
Α3-15	A3	0	0	0	0	**0**
B1-1	B1	0	0	0	0	**0**
B1-2	B1	0	1	0	0	**1**
B1-3	B1	0	1	1	1	**3**
B1-4	B1	0	0	0	0	**0**
B1-5	B1	0	1	0	0	**1**
B2-6	B2	0	0	0	0	**0**
B2-7	B2	0	0	0	0	**0**
B2-8	B2	0	0	0	0	**0**
B2-9	B2	0	0	0	0	**0**
B2-10	B2	0	0	0	0	**0**
B3-11	B3	0	0	0	0	**0**
B3-12	B3	0	0	0	0	**0**
B3-13	B3	0	0	0	0	**0**
B3-14	B3	0	0	0	0	**0**
B3-15	B3	0	0	0	0	**0**
C1-1	C1	0	0	0	0	**0**
C1-2	C1	0	1	0	0	**1**
C1-3	C1	1	0	1	1	**3**
C1-4	C1	1	2	2	1	**6**
C1-5	C1	0	2	2	1	**5**
C2-6	C2	0	0	0	0	**0**
C2-7	C2	0	0	0	0	**0**
C2-8	C2	0	0	0	0	**0**
C2-9	C2	0	0	0	0	**0**
C2-10	C2	0	0	0	0	**0**
C3-11	C3	1	0	0	1	**2**
C3-12	C3	0	1	0	0	**1**
C3-13	C3	0	0	0	0	**0**
C3-14	C3	1	0	0	1	**2**
C3-15	C3	0	0	0	0	**0**
D1-1	D1	0	0	0	0	**0**
D1-2	D1	0	1	0	0	**1**
D1-3	D1	1	1	1	1	**4**
D1-4	D1	1	0	1	1	**3**
D1-5	D1	0	0	0	0	**0**
D2-6	D2	0	1	0	0	**1**
D2-7	D2	0	0	1	0	**1**
D2-8	D2	0	1	0	0	**1**
D2-9	D2	0	0	0	0	**0**
D2-10	D2	0	0	0	0	**0**
D3-11	D3	0	0	0	0	**0**
D3-12	D3	0	1	0	0	**1**
D3-13	D3	0	0	0	0	**0**
D3-14	D3	0	0	0	0	**0**
D3-15	D3	0	0	0	0	**0**

Liver histological analysis for the following parameters: sinusoidal congestion, vacuolization of hepatocyte cytoplasm, parenchymal necrosis, and inflammation, with the total score representing the sum of all parameters for each individual animal. Each parameter was graded numerically as follows: congestion, vacuolization, and inflammation: 0 = none, 1 = minimal, 2 = mild, 3 = moderate, and 4 = severe. The numerical graduation for necrosis was as follows: 0 = nonnecrotic cells, 1 = single-cell necrosis, 2 ≤ 30% necrosis, 3 ≤ 60% necrosis, and 4 ≥  60% necrosis. ^1^The subgroup ID is depicted in Table 1 (experimental design). ^2^Total score represents the sum of all parameters for each individual animal.

**Table 4:** Serum values of aspartate aminotransferase, alanine aminotransferase, and number of platelets.

Aspartate aminotransferase (AST)						Alanine aminotransferase (ALT)						Platelet number (PLT) (x1000)					
Subgroup ID^1^	Number of animals	Mean	Standard deviation	95% confidence interval for mean		Subgroup ID	Number of animals	Mean	Standard deviation	95% confidence interval for mean		Subgroup ID	Number of animals	Mean	Standard deviation	95% confidence interval for mean	
				Lower bound	Upper bound					Lower bound	Upper bound					Lower bound	Upper bound
4th postoperative day of sacrifice																	
CN1	5	164.80	27.004	131.27	198.33	CN1	5	65.00	24.197	34.96	95.04	CN1	5	939.40	123.253	786.36	1092.44
S1	5	106.00	31.401	67.01	144.99	S1	5	41.00	6.892	32.44	49.56	S1	5	1022.60	132.657	857.88	1187.32
A1	5	1646.60	3457.812	−2646.84	5940.04	A1	5	884.80	1911.953	−1489.20	3258.80	A1	5	808.20	221.719	532.90	1083.50
B1	5	123.60	44.909	67.84	179.36	B1	5	47.80	18.404	24.95	70.65	B1	5	1044.20	86.757	936.48	1151.92
C1	5	125.60	24.785	94.83	156.37	C1	5	57.20	16.022	37.31	77.09	C1	5	839.00	134.030	672.58	1005.42
D1	5	154.80	70.183	67.66	241.94	D1	5	43.60	10.502	30.56	56.64	D1	5	1016.20	132.315	851.91	1180.49
7th postoperative day of sacrifice																	
CN2	5	619.80	1069.440	−708.08	1947.68	CN2	5	83.20	54.642	15.35	151.05	CN2	5	948.20	141.111	772.99	1123.41
S2	5	107.80	27.725	73.37	142.23	S2	5	53.00	16.263	32.81	73.19	S2	5	1028.20	78.004	931.34	1125.06
A2	5	146.60	40.692	96.07	197.13	A2	5	41.80	13.737	24.74	58.86	A2	5	1006.00	96.946	885.63	1126.37
B2	5	107.60	48.588	47.27	167.93	B2	5	53.00	22.869	24.60	81.40	B2	5	1044.80	226.942	763.01	1326.59
C2	5	164.00	142.367	−12.77	340.77	C2	5	70.60	37.038	24.61	116.59	C2	5	854.80	237.579	559.81	1149.79
D2	5	116.80	12.050	101.84	131.76	D2	5	52.80	7.396	43.62	61.98	D2	5	1059.40	178.106	838.25	1280.55
15th postoperative day of sacrifice																	
CN3	5	127.40	49.772	65.60	189.20	CN3	5	45.80	14.618	27.65	63.95	CN3	5	675.00	249.933	364.67	985.33
S3	5	99.00	38.216	51.55	146.45	S3	5	42.60	7.765	32.96	52.24	S3	5	772.60	83.395	669.05	876.15
A3	5	99.00	24.197	68.96	129.04	A3	5	45.40	20.428	20.04	70.76	A3	5	625.60	91.172	512.40	738.80
B3	5	150.40	48.418	90.28	210.52	B3	5	63.20	10.569	50.08	76.32	B3	5	909.40	47.221	850.77	968.03
C3	5	148.38	52.407	83.31	213.45	C3	5	44.38	6.485	36.33	52.43	C3	5	813.40	278.302	467.84	1158.96
D3	5	242.60	99.354	119.24	365.96	D3	5	60.80	20.499	35.35	86.25	D3	5	914.40	56.801	843.87	984.93

Demonstrating the mean serum values of aspartate aminotransferase, alanine aminotransferase, and the number of platelets, as well as the standard deviation and the lower and upper bound of the 95% confidence interval for the mean value, in each experimental subgroup. ^1^The subgroup ID is depicted in Table 1 (experimental design).

**Table 5:** AST, ALB, PT, and INR statistical significant differences.

Aspartate aminotransferase (AST)				Albumin (ALB)				Prothrombin time (PT)				International normalized ratio (INR)			
Subgroup ID number (I)^1^	Subgroup ID number (J)^1^	Mean difference (I–J)	Statistical significance (*p* value^2^)	Subgroup ID number (I)	Subgroup ID number (J)	Mean difference (I–J)	Statistical significance (*p* value)	Subgroup ID number (I)	Subgroup ID number (J)	Mean difference (I–J)	Statistical significance (*p* value)	Subgroup ID number (I)	Subgroup ID number (J)	Mean difference (I–J)	Statistical significance (*p* value)
D_3_	N	−134.600	0.010	N	B_1_	1.9600	0.000	N	CN_1_	−1.79600	0.0001	N	CN_1_	−.14600	0.0001
	S_3_	143.600	0.008		C_1_	1.2000	0.020		A_1_	−1.01600	0.0001		A_1_	−0.08600	0.0001
	A_3_	143.600	0.008	CN_1_	B_1_	1.3800	0.005		B_1_	−0.95600	0.0001		B_1_	−0.07600	0.0001
				S_1_	B_1_	1.7600	0.000		C_1_	−0.97400	0.0001		C_1_	−0.07600	0.0001
				B_1_	D_1_	−1.2200	0.017		D_1_	−0.91600	0.0001		D_1_	−0.07600	0.0001
				N	CN_2_	0.5200	0.004	CN_1_	S_1_	1.92000	0.0001	CN_1_	S_1_	0.15600	0.0001
					A_2_	0.5000	0.007		A_1_	0.78000	0.0001		A_1_	0.06000	0.0001
					C_2_	0.9400	0.0001		B_1_	0.84000	0.0001		B_1_	0.07000	0.0001
					D_2_	0.5800	0.001		C_1_	0.82200	0.0001		C_1_	0.07000	0.0001
				CN_2_	S_2_	−0.4400	0.028		D_1_	0.88000	0.0001		D_1_	0.07000	0.0001
					C_2_	0.4200	0.042	S_1_	A_1_	−1.14000	0.0001	S_1_	A_1_	−0.09600	0.0001
				S_2_	A_2_	0.4200	0.042		B_1_	−1.08000	0.0001		B_1_	−0.08600	0.0001
					C_2_	0.8600	0.000		C_1_	−1.09800	0.0001		C_1_	−0.08600	0.0001
					D_2_	0.5000	0.009		D_1_	−1.04000	0.0001		D_1_	−0.08600	0.0001
				A_2_	C_2_	0.4400	0.028	CN_2_	N	−1.03400	0.0001	CN_2_	N	−0.08400	0.0001
				B_2_	C_2_	0.5800	0.002		S_2_	1.08200	0.0001		S_2_	0.08800	0.000
				N	A_3_	1.5400	0.016		A_2_	0.73800	0.001		A_2_	0.06000	0.001
					B_3_	3.1400	0.0001		B_2_	0.77800	0.001		B_2_	0.06000	0.001
				B_3_	CN_3_	−2.8400	0.000		C_2_	0.75600	0.001		C_2_	0.06000	0.001
					S_3_	−3.0200	0.000		D_2_	0.80800	0.000		D_2_	0.07000	0.000
					A_3_	−1.6000	0.019	CN_3_	N	−0.76200	0.001	CN_3_	N	−0.06200	0.001
					C_3_	−2.8400	0.000		S_3_	0.81200	0.000		S_3_	0.06600	0.000
					D_3_	−2.4600	0.000		A_3_	0.71800	0.000		A_3_	0.06000	0.000
									B_3_	0.76200	0.000		B_3_	0.06200	0.000
									C_3_	0.74800	0.000		C_3_	0.06000	0.000
									D_3_	0.79200	0.000		D_3_	0.06800	0.000

Statistically significant differences between different experimental subgroups, regarding the serum levels of aspartate aminotransferase, albumin, prothrombin time, and international normalized ratio. ^1^The subgroup ID is depicted in Table 1 (experimental design). ^2^The level of statistical significance was set at 5% (*α* = 0.05).

**Table 6:** GGT, ALP, and TBIL statistical significant differences.

Gamma-glutamyltransferase (GGT)				Alkaline phosphatase (ALP)				Total bilirubin (TBIL)			
Subgroup ID number (I)^1^	Subgroup ID number (J)^1^	Mean difference (I–J)	Statistical significance (*p* value^2^)	Subgroup ID number (I)	Subgroup ID number (J)	Mean difference (I–J)	Statistical significance (*p* value)	Subgroup ID number (I)	Subgroup ID number (J)	Mean difference (I–J)	Statistical significance (*p* value)
D_1_	B_1_	3.720	0.007	B_1_	S_1_	140.000	0.048	CN_1_	A_1_	−0.15800	0.012
	C_1_	3.200	0.032	D_2_	N	153.800	0.014	A_1_	S_1_	0.15800	0.012
D_3_	N	4.800	0.003						C_1_	0.14800	0.023
	S_3_	4.200	0.014					CN_2_	N	0.10600	0.006
	A_3_	5.520	0.001						S_2_	0.09200	0.024
	B_3_	5.760	0.000						A_2_	0.10000	0.010
	C_3_	5.180	0.001						B_2_	0.10800	0.004
									C_2_	0.11800	0.002
									D_2_	0.10800	0.004
								CN_3_	N	0.15600	0.001
									S_3_	0.16800	0.000
									A_3_	0.12000	0.024
								C_3_	S_3_	0.12200	0.021
											
											

Statistically significant differences between different experimental subgroups, regarding the serum levels of Gamma-glutamyltransferase, alkaline phosphatase, and total bilirubin. The level of statistical significance was set at 5% (*α* = 0.05). ^1^The subgroup ID is depicted in Table 1 (experimental design).

**Table 7:** DBIL, IBIL, and PR statistical significant differences.

Direct bilirubin (DBIL)				Indirect bilirubin (IBIL)				Total proteins (PR)			
Subgroup ID number (I)	Subgroup ID number (J)	Mean difference (I–J)	Statistical significance (*p* value)	Subgroup ID number (I)	Subgroup ID number (J)	Mean difference (I–J)	Statistical significance (*p* value)	Subgroup ID number (I)	Subgroup ID number (J)	Mean difference (I–J)	Statistical significance (*p* value)
A_1_	N	0.09400	0.014	C_3_	N	0.10000	0.001	N	A_1_	1.1400	0.003
	CN_1_	0.09800	0.009		S_3_	0.10200	0.000		B_1_	1.3000	0.001
	S_1_	0.09800	0.009		A_3_	0.09000	0.002		C_1_	1.2800	0.001
	C_1_	0.09800	0.009		B_3_	0.07800	0.011		D_1_	1.0600	0.007
CN_2_	N	0.07600	0.009	D_3_	N	0.08600	0.004	S_1_	B_1_	0.8800	0.042
	S_2_	0.07000	0.021		S_3_	0.08800	0.003	N	CN_2_	0.9200	0.006
	A_2_	0.06400	0.048		A_3_	0.07600	0.014		A_2_	0.8200	0.019
	B_2_	0.07400	0.012						B_2_	1.0800	0.001
	C_2_	0.08400	0.003						C_2_	0.9600	0.004
	D_2_	0.08200	0.004						D_2_	1.2000	0.0001
CN_3_	N	0.10800	0.0001					N	CN_3_	1.0000	0.004
	S_3_	0.11800	0.000						A_3_	1.0400	0.002
	A_3_	0.08200	0.016					B_3_	CN_3_	1.0600	0.002
	C_3_	0.09800	0.002						A_3_	1.1000	0.001
	D_3_	0.11000	0.000								

Statistically significant differences between different experimental subgroups, regarding the serum levels of direct bilirubin, indirect bilirubin, and total proteins. The level of statistical significance was set at 5% (*α* = 0.05). The subgroup ID is depicted in Table 1 (experimental design).
